# Identification of independent predictors of successful external cephalic version: A retrospective multicenter study

**DOI:** 10.1097/MD.0000000000050888

**Published:** 2026-10-02

**Authors:** Yali Yang, Qiaofang Yang, Xufei Fan

**Affiliations:** a Department of Obstetrics, Jinhua Maternal and Child Health Care Hospital, Jinhua, Zhejiang, China; b Department of Obstetrics, Jinhua Municipal Central Hospital, Jinhua, Zhejiang, China; c Department of Obstetrics, Jinhua’s Key Laboratory of Comprehensive Prevention and Treatment of Birth Defects, Jinhua, Zhejiang, China.

**Keywords:** breech presentation, cesarean delivery, external cephalic version, predictors, success rate

## Abstract

This study aimed to determine the success rate of external cephalic version (ECV) and identify independent predictors of successful ECV among women with a term singleton fetus in breech presentation. This retrospective multicenter study included 65 pregnant women who underwent ECV at 2 centers between January 2017 and January 2024. Participants were categorized into successful (n = 39) and failed (n = 26) ECV groups. Univariate analyses and multivariable logistic regression were used to identify independent predictors of successful ECV. The overall ECV success rate was 60.0% (39/65). Multivariable analysis showed that multiparity (odds ratio [OR], 5.54; 95% confidence interval [CI], 1.52–20.12; *P* = .009) and a nonanterior placental location (OR, 4.72; 95% CI, 1.31–17.03; *P* = .018) were independent positive predictors of ECV success. Maternal body mass index showed a borderline inverse association with success (OR, 0.84; 95% CI, 0.70–1.00; *P* = .053). No severe maternal or neonatal complications occurred. Vaginal delivery was achieved by 89.7% of women in the successful ECV group and none in the failed ECV group (*P* < .001). Multi parity and a nonanterior placental location were associated with greater odds of successful ECV. Pre procedural assessment of these factors may support individualized counseling and clinical decision-making aimed at reducing cesarean delivery rates.

## 1. Introduction

At term, breech presentation remains the most common fetal mal presentation, complicating nearly 3% to 4% of all term deliveries.^[1]^ While spontaneous version typically occurs before 32 weeks of gestation, the persistence of a breech position toward term significantly elevates the risk of obstetric emergencies, including premature rupture of membranes, umbilical cord prolapse, and neonatal asphyxia.^[2,3]^ Historically, the management of term breech has shifted toward elective cesarean section (CS) to mitigate birth trauma and dystocia. However, the global surge in CS rates has introduced secondary challenges such as increased maternal morbidity and complications in subsequent pregnancies.^[4]^

External cephalic version is a transabdominal manual maneuver used to rotate a fetus from breech to cephalic presentation. It is an important noninvasive strategy for reducing cesarean delivery rates and increasing the likelihood of vaginal birth.^[1,5]^ Despite these benefits, external cephalic version (ECV) success rates vary and depend on a complex combination of maternal, fetal, and procedural characteristics.^[6]^

Reported ECV success rates range from 50% to 70%, and identifying reliable independent predictors remains an important focus of perinatal research.^[6,7]^ Accurate pre procedural estimation may improve individualized counseling, clinical decision-making, and procedural planning. We therefore analyzed data from 65 patients treated at 2 obstetric centers to identify independent predictors of ECV success and evaluate procedural safety.

## 2. Materials and methods

### 2.1. Study population

We retrospectively reviewed 65 women with a term singleton fetus in breech presentation who underwent ECV at 2 obstetric centers (Jinhua Municipal Central Hospital and Jinhua Maternal and Child Health Care Hospital) between January 2017 and January 2024. This study was approved by the Ethics Committee of Jinhua Maternal and Child Health Care Hospital (approval No. 2025QT002). The requirement for informed consent was waived because of the retrospective study design and the use of anonymized data.

To minimize selection bias, all consecutive cases during the study period were initially screened for eligibility. As this was a retrospective analysis of all available consecutive patients, a formal sample size calculation was not performed prior to the study; the final sample size (n = 65) was determined by the number of cases that met all eligibility criteria.

Inclusion criteria:

• Singleton gestation.
• Breech presentation confirmed by ultrasound.
• Gestational age 36 to 39 weeks.

Exclusion criteria:

• Placenta previa.
• Placental abruption.
• Uterine scar.
• Signs of labor.

Based on the immediate outcome of the ECV procedure, the final included subjects were divided into 2 cohorts: the success group (n = 39) and the failure group (n = 26).

### 2.2. Procedure

All procedures were performed by senior obstetricians under continuous ultrasonographic and fetal heart rate monitoring. Patients were placed supine with their hips slightly elevated. Tocolytic agents (e.g., ritodrine) were administered selectively according to uterine tone. After the procedure, fetal heart rate monitoring was continued; when ECV was successful, a compression bandage was applied.

### 2.3. Statistical analysis

All statistical analyses were performed using SPSS version 26.0 (IBM Corp., Armonk, NY). Continuous variables are presented as the mean ± standard deviation and were compared using independent-samples *t* tests. Categorical variables are presented as number (percentage) and were compared using the chi-square test or Fisher exact test, as appropriate.

Variables associated with ECV success in the univariate analysis were entered into a multivariable logistic regression model to identify independent predictors and account for potential confounding. Parity and placental location were evaluated as candidate predictors; maternal body mass index, amniotic fluid index, and tocolytic use were included as covariates.

Missing data: Electronic medical records were reviewed for all screened patients. Patients with missing primary outcome or critical baseline data were excluded during screening, as shown in the participant flow diagram. Therefore, no imputation was performed for the final analytic cohort (n = 65).

## 3. Results

### 3.1. Participant flow

A total of 83 pregnant women with a term singleton fetus in breech presentation who underwent an ECV attempt were screened. Eighteen were excluded because delivery outcome data were unavailable after delivery at other hospitals. The final retrospective cohort therefore comprised 65 patients, categorized according to the immediate procedural outcome as successful ECV (n = 39) or failed ECV (n = 26) (Fig. 1). The overall success rate was 60.0% (39/65). Baseline characteristics and univariate analyses are presented in Table 1. Amniotic fluid index (*P* = .039), parity (*P* = .016), and placental location (*P* = .010) were significantly associated with ECV success; maternal body mass index showed a borderline association (*P* = .053).

**Table 1:** Baseline clinical characteristics and univariate analysis.

Variables	Success group (n = 39)	Failure group (n = 26)	*P* value
Age (yr)	30.4 ± 4.9	29.9 ± 4.1	.652
Gestational age at ECV (wk)	37.1 ± 1.3	36.9 ± 1.1	.664
Maternal BMI (kg/m2)	26.8 ± 3.5	28.6 ± 3.5	.053
Amniotic fluid index (mm)	132.9 ± 35.1	116.3 ± 28.1	.039*
Multi parity, n (%)	28 (71.8%)	10 (38.5%)	.016*
Non-anterior placenta, n (%)	30 (76.9%)	11 (42.3%)	.010*
Use of tocolytics, n (%)	24 (61.5%)	20 (76.9%)	.304

Continuous and categorical variables are presented as mean ± SD and n (%), respectively. Statistical significance was set at *P* < .05.

BMI = body mass index, ECV = external cephalic version.

**Figure 1.**
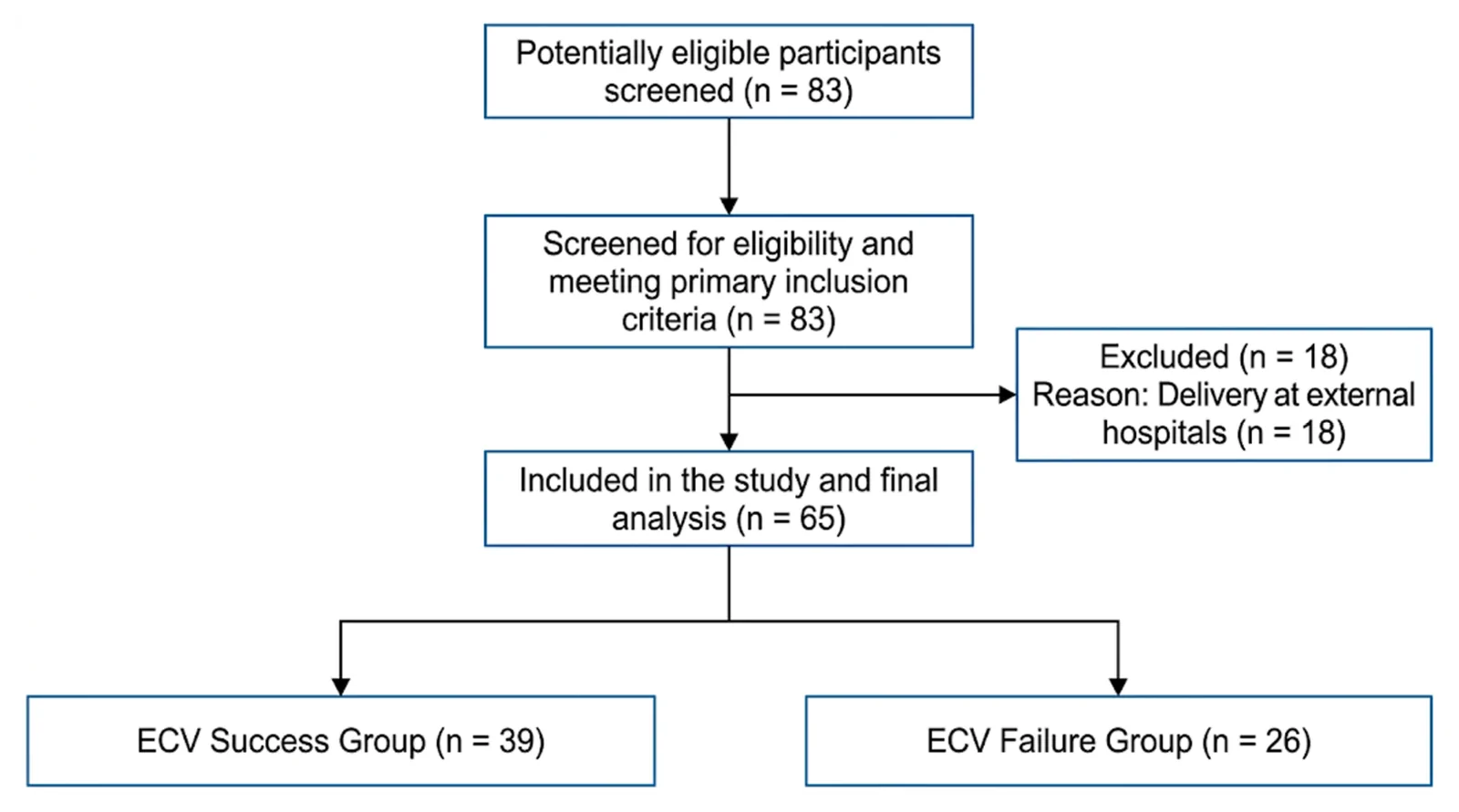
Flowchart of patient selection process. ECV = external cephalic version.

Multivariate logistic regression confirmed that multi parity and non-anterior placental location were strong independent predictors of a successful version (Table 2).

**Table 2:** Multivariate logistic regression analysis for predictors of ECV success.

Variables	Odds ratio (OR)	95% CI	*P* value
Parity (multi vs Nulli)	5.54	1.52–20.12	.009*
Placental location (non-ant vs ant)	4.72	1.31–17.03	.018*
Maternal BMI	0.84	0.70–1.00	.053

Statistical significance was defined as *P* < .05. Tocolytic administration and amniotic fluid index were included as covariates in the multivariate analysis, but were not significant.

Ant = anterior, BMI = body mass index, CI = confidence interval, Multi = multiparous, Nulli = nulliparous, OR = odds ratio.

Delivery outcomes are summarized in Table 3. Vaginal delivery occurred in 89.7% of the successful ECV group and 0% of the failed ECV group (*P* < .001). All patients in the failed ECV group underwent cesarean delivery. Estimated postpartum blood loss did not differ significantly between groups (*P* = .548).

**Table 3:** Comparison of maternal and neonatal outcomes.

Outcomes	Success group (n = 39)	Failure group (n = 26)	*P* value
Gestational age at delivery (wk)	38.8 ± 1.5	37.9 ± 1.0	.004*
Vaginal delivery, n (%)	35 (89.7%)	0 (0.0%)	<.001*
Cesarean section, n (%)	4 (10.3%)	26 (100.0%)	<.001*
Postpartum hemorrhage (mL)	290.3 ± 137.3	273.1 ± 91.9	.548

* *P* < .05 indicates statistical significance.

## 4. Discussion

Breech presentation remains a common indication for primary cesarean delivery worldwide. Reducing the number of cesarean births associated with breech presentation remains an important goal in routine obstetric care. In the present study, the overall success rate of ECV was 60.0%, which is consistent with the 50% to 70% success rate reported in previous clinical studies.^[4,8]^ Our data confirm that ECV is a clinically valuable procedure and further identifies multi parity and non-anterior placental location as 2 powerful independent predictors of a successful version.^[6,7]^

Parity exerted a strong influence on ECV outcomes in this study. Multiparous participants were 5.5 times more likely to achieve a successful version than nulliparous women (odds ratio = 5.54, *P* = .009). This difference is commonly observed in clinical practice and may be explained by looser abdominal wall tension and greater uterine pliability among women with prior vaginal deliveries. Such anatomical characteristics reduce mechanical resistance and allow for easier manipulation during fetal rotation.^[6,7]^ In contrast, nulliparous women typically have a taut abdominal wall and a higher baseline uterine tone, which can hinder rotation and increase the risk of procedural failure.

Placental position also affected procedural success. A non-anterior placenta was associated with a 4.7-fold higher likelihood of a successful ECV (odds ratio = 4.72, *P* = .018). The anterior placenta lies directly under the abdominal wall and acts as a physical cushion during manual rotation. This layer may dampen the force applied by the operator and limit accurate contact with fetal parts, making controlled rotation more difficult.^[5,9]^ These findings support the value of routine prenatal ultrasound in documenting placental location before ECV is attempted. Such information helps clinicians to adjust techniques and provide realistic prognostic information to patients.

An unexpected finding in this study was the higher rate of tocolytic use among the women in the failure group. Although several studies have suggested that tocolytic agents may improve ECV success,^[10]^ our data did not show such a benefit. This discrepancy is likely due to confounding by indication in clinical practice; tocolytics were more frequently administered in difficult cases with increased uterine tone or early resistance to rotation. Thus, tocolytic use reflects case complexity, rather than treatment failure. This observation highlights the need for consistent protocols rather than selective use of tocolysis during ECV.

Maternal body mass index showed a borderline inverse association with ECV success (*P* = .053^[9]^ A higher body mass index increases the thickness of subcutaneous fat tissue in the abdominal wall. This layer can reduce the precision of fetal palpation and interfere with the transmission of rotational force, making the procedure more challenging.

ECV was safe in this cohort, with no severe maternal or neonatal complications, and no significant difference in postpartum hemorrhage between the groups. The strongest clinical finding was the effect of ECV on the delivery route: all women with failed ECV underwent CS, while nearly 90% of those with successful ECV delivered vaginally. This marked difference reinforces the role of ECV as an effective strategy to lower cesarean rates in term breech pregnancies.^[1,2]^

This study has several strengths. First, inclusion of consecutive patients from 2 obstetric centers may improve the representativeness of the cohort relative to a single-center series. Second, the 7-year study period provided a longitudinal clinical sample in which routinely available predictors could be evaluated. Third, the identified factors parity and placental location are readily assessed before ECV and may help clinicians counsel patients and plan procedures. These strengths should nevertheless be interpreted in the context of the retrospective design and modest sample size.

This study also has limitations. Its retrospective design carries an inherent risk of selection bias. In particular, 18 patients who underwent ECV at the participating centers were excluded because they subsequently delivered elsewhere and lacked delivery outcome data; this exclusion may have biased estimates of delivery mode and neonatal outcomes. In addition, the final sample of 65 patients across 2 centers provided limited statistical power to detect weaker associations, such as the borderline association with maternal body mass index (*P* = .053), and produced wide confidence intervals for the principal predictors. Larger prospective multicenter studies using standardized ECV protocols are needed to validate these findings.

## 5. Conclusion

Multi parity and a nonanterior placental location were independently associated with successful ECV. Assessing these factors before the procedure may support individualized counseling and clinical decision-making and may help reduce cesarean delivery rates.

## Author contributions

**Data curation:** Yali Yang, Qiaofang Yang.

**Formal analysis:** Yali Yang.

**Funding acquisition:** Yali Yang.

**Conceptualization:** Xufei Fan.

**Methodology:** Xufei Fan.

**Writing – original draft:** Yali Yang, Xufei Fan.

**Writing – review & editing:** Yali Yang, Qiaofang Yang, Xufei Fan.
